# Prediction of clinical outcome in patients treated with cardiac resynchronization therapy - the role of NT-ProBNP and a combined response score

**DOI:** 10.1186/s12872-018-0802-8

**Published:** 2018-04-24

**Authors:** Z. Bakos, N. C. Chatterjee, C. Reitan, J. P. Singh, R. Borgquist

**Affiliations:** 1 Department of Clinical Sciences, Arrhythmia section, Lund University, Skane University Hospital, Lund, Sweden; 2 Division of Cardiology, Massachusetts General Hospital, Boston, USA

**Keywords:** Cardiac resynchronization therapy, Heart failure, Dilated cardiomyopathy, Cardiac response, NT-proBNP

## Abstract

**Background:**

Cardiac resynchronization therapy (CRT) is an established therapy for appropriately selected patients with heart failure. Response to CRT has been heterogeneously defined using both clinical and echocardiographic measures, with poor correlation between the two.

**Methods:**

The study cohort was comprised of 202 CRT-treated patients and CRT response was defined at 6 months post-implant. Echocardiographic response (E+) was defined as a reduction in LVESV ≥ 15%, clinical response as an improvement of ≥ 1 NYHA class (C+), and biomarker response as a ≥ 25% reduction in NT-proBNP(B+). The association of response measures (E+, B+, C+; response score range 0–3) and clinical endpoints at 3 years was assessed in landmarked Cox models.

**Results:**

Echo and clinical responders demonstrated greater declines in NT-proBNP than non-responders (median [E+/B+]: -52%, [E+]: -27%, [C+]: -39% and [E-/C-]: -13%; *p* = 0.01 for trend). Biomarker (HR 0.43 [95% CI: 0.22–0.86], *p* = 0.02) and clinical (HR 0.40 [0.23–0.70] *p* = 0.001) response were associated with a significantly reduced risk of the primary endpoint. When integrating each response measure into a composite score, each 1 point increase was associated with a 31% decreased risk for a composite endpoint of mortality, LVAD, transplant and HF hospitalization (HR 0.69 [95% CI: 0.50–0.96], *p* = 0.03), and a 52% decreased risk of all-cause mortality (HR 0.48 [95% CI: 0.26–0.89], *p* = 0.02).

**Conclusion:**

Serial changes in NT-proBNP are associated with clinical outcomes following CRT implant. Integration of biomarker, clinical, and echocardiographic response may discriminate CRT responders versus non-responders in a clinically meaningful way, and with higher accuracy.

**Trial registration:**

The cohort was combined from study NCT01949246 and the study based on local review board approval 2011/550 in Lund, Sweden.

**Electronic supplementary material:**

The online version of this article (10.1186/s12872-018-0802-8) contains supplementary material, which is available to authorized users.

## Background

Cardiac resynchronization therapy (CRT) is a well-validated treatment for morbidity and mortality reduction in select patients with heart failure [1–4]. However, non-response to cardiac resynchronization therapy (CRT) continues to be a clinical challenge, and the cause is likely multifactorial [5]. It may involve one or several factors such as suboptimal left ventricular lead positioning, suboptimal device programming, myocardial scarring next to the pacing electrodes, absence of “correctable dyssynchrony” to start with, or aggravating concurrent comorbid conditions such as renal failure, iron deficiency and anemia [6–8]. Other comorbidities such as diabetes and the metabolic syndrome, have also been reported to have an influence on the clinical outcome of CRT results [9]. Using advanced CRT technology can offer improvement in this population [10]. Indeed, there is good evidence that a structured “non-responder” outpatient clinic may identify and address several of these issues, thereby likely improving the clinical outcome [11, 12]. However, defining “positive response” has proven challenging, with heterogeneous definitions including echocardiographic and clinical response. In addition, correlation between echocardiographic and clinical response has been poor. [13, 14]. In this context, biochemical markers such as natriuretic peptides (for instance NT-proBNP) and micro-RNA have been shown to be associated with both clinical and structural measures in HF, and may help refine the classification of response to CRT and improve prognostication [15–17]. Heart failure leads affects the neuro-hormonal balance and induces changes on the molecular level. MicroRNA is a novel marker which may be associated with cardiovascular disease, such as arrhythmias or HF, and there are trials suggesting that CRT treatment induced reverse remodelling may be associated with reduction of the microRNA expression [17].

In this study however, we focused on examining the relationship between clinical, echocardiographic, and natriuretic peptide response to CRT. We then examined the association of an integrated response score with long-term clinical outcomes.

## Methods

Four hundred and-ten consecutive patients eligible for CRT therapy were prospectively recruited from two tertiary referral centers; Massachusetts General Hospital in Boston, USA and Skane University Hospital in Lund, Sweden between 2011 and 2014. Of these patients nine died prior to follow up, and only 202 had paired echocardiographic, clinical, and biomarker data available; therefore 208 patients were excluded from further analysis. Patients with a class I or class II indication for CRT according to the guideline recommendations were included [18, 19]. All patients underwent standard CRT implant procedure with transvenous leads. LV leads were positioned in the most favorable available anatomic branch of the coronary sinus (CS) that resulted in adequate lead stability with acceptable pacing parameters, without diaphragmatic stimulation. Mortality data was extracted from the Swedish national registry of death or from the United States Social Security Death Index. Clinical events including heart failure hospitalizations, heart transplantation, left ventricular assist devices (LVAD) and mortality were ascertained via chart review. Heart failure hospitalization was defined as an inpatient admission because of acute cardiac decompensation, with improvement of symptoms after adequate heart failure treatment. The primary endpoint was a composite of all-cause death, heart transplantation, left ventricular assist device (LVAD), and heart failure hospitalization. The study conforms to the Declaration of Helsinki, and the study protocols were approved by the respective Institutional Review Board and Ethics committee. All patients signed written informed consent prior to enrollment.

### Echocardiography

The echocardiographic studies were performed with a standard imaging system (Vivid E9, GE Medical, Horten, Norway). Offline analysis was performed on a PC workstation with Echopac software (Echopac BT12, GE Medical, Horten, Norway). Standard echocardiographic assessment was performed in all patients preoperative and 6 month after the implantation. ft. ventricular end-diastolic volume (LVEDV) and left ventricular end-systolic volume (LVESV) were measured, and left ventricular end systolic volume index was calculated (LVESVi).

### Definition of CRT response

*Echocardiographic response* was defined as a reduction of LVESV ≥15% [20]. The *clinical response* was based on the New York Heart Association (NYHA) classification and was considered positive if the NYHA improvement was ≥1 class [14] from pre-implant level. *Dual responders* were those individuals who had objective improvement on the echocardiography and at the same time the NYHA class improved with more than one class. Patients who did not fulfill definitions of clinical or echo response were classified as *non-responders*. NT-proBNP was measured at baseline and at 6 months post-implant using standard commercial assays, and in paired analysis a ≥ 25% relative reduction of the baseline NT-proBNP level was considered as a positive biomarker response.

### Statistical analyses

SPSS statistical software was used for all data analysis (IBM corp. SPSS ver. 22, 2014). Continuous variables are expressed as mean (SD, standard deviation) or median (IQR, interquartile range) as appropriate, categorical variables are presented as frequencies and percentages. Differences between groups were assessed using Student t-tests for continuous variables, Mann Whitney U test for variables with non-normal distribution, and the Fisher’s exact test or Chi^2^ test or for categorical variables as appropriate. Paired T-test was used to compare echocardiographic dimensions from baseline to follow-up. The Wilcoxon paired Signed Rank test was used to assess changes in NT-proBNP levels and NYHA class. Cox regression analysis was used for prediction of the clinical endpoints, first in univariate analysis, and then in a pre-specified multivariate model adjusted for the clinically relevant co-variables age, sex, renal disease, ECG morphology and etiology of heart failure [21–23].

For identification of the proper cut off level of the relative NT-proBNP change, ROC analysis was performed in each subgroup. The goal was to identify a cut-off with highest possible sensitivity yet acceptable specificity, to differentiate the non-responders from responders. Survival analysis using the Kaplan-Meier method with log-rank test was used to analyze the cumulative events with a landmark set at 6 month, excluding all events prior to that. A two-sided *p*-value < 0.05 was considered statistically significant.

## Results

The participants -similarly to other randomized CRT trials- were dominantly male (78%) in the age of 70’s with ischemic CMP (50%) and LBBB(64%) with mean EF of 27 ± 7%. Baseline characteristics in details for the study cohort (*N* = 202) are shown in Table 1**.** Over a median follow-up of 1133 (518 IQR) days, there were 48 patients who experienced at least one component of the composite endpoint, including 38 heart failure hospitalizations, 0 LVAD implantations, 0 transplants, and 25 deaths. Only 22% of patients were women, and there was no difference regarding clinical outcome or NT-ProBNP compared to male patients, however women were more likely to have a reduction of ESV ≥15% (*p* = 0.04).

**Table 1:** Study Population characteristics, stratified by BNP reduction ≥25%, with *p*-value for difference between groups

	All patients (*n* = 202)	BNP reduction < 25% (*n* = 78)	BNP reduction ≥ 25% (*n* = 124)	*P*-value
Male gender	157 (78%)	61 (78%)	96 (77%)	1.0
Age, (years) (median IQR)	71 (14)	72 (14)	69 (13)	0.14
LV ejection fraction (mean ± SD)	27 ± 7	27 ± 6	27 ± 8	0.92
LVESV (mean ± SD)	158 ± 68	148 ± 73	164 ± 65	0.09
QRS duration (ms, median IQR)	161 ± 23	154 ± 23	166 ± 22	< 0.001
Ischemic cardiomyopathy	100 (50%)	38 (49%)	62 (50%)	0.89
Hypertension	121 (60%)	47 (60%)	74 (60%)	1.0
Diabetes	50 (25%)	21 (27%)	29 (23%)	0.62
Atrial Fibrillation	91 (45%)	37 (47%)	54 (44%)	0.66
Previous CABG	52 (26%)	22 (28%)	30 (24%)	0.62
LBBB	129 (64%)	42 (54%)	87 (70%)	0.02
LBBB > 150 msec	148 (73%)	46 (59%)	102 (82%)	0.01
NYHA class III-IV	151 (75%)	55 (71%)	96 (77%)	0.32
ACEi or ARB use	179 (89%)	69 (89%)	110 (89%)	1.0
Loop diuretic use	147 (73%)	60 (77%)	87 (70%)	0.33
Anticoagulant use	91 (45%)	34 (44%)	57 (46%)	0.77
Digoxin use	27 (13%)	12 (15%)	15 (12%)	0.53
Beta-blocker use	176 (87%)	65 (83%)	111 (90%)	0.28
Creatinine pre implant (mg/dl)	1.12 (0.45)	1.20 (0.52)	1.08 (0.44)	0.05
CRT-D vs. CRT-Pacemaker	169 (84%)	63 (81%)	106 (86%)	0.44
NT-proBNP baseline (ng/L median IQR)	1554 (3393)	1444 (3770)	1661 (2968)	0.31

*CABG* coronary artery bypass surgery, *CRT-D* cardiac resynchronization therapy with defibrillator function, *LBBB* left bundle branch block, *NYHA* New York Heart Association, *ACEi* ACE-inhibitor, *ARB* angiotensin receptor blocker

### Parameters for prediction of clinical outcome

From baseline to 6 months, mean left ventricular ejection fraction increased from 27 ± 7% to 34 ± 9%, LV end-systolic volume decreased from 158 ± 69 ml to 124 ± 67 ml and LV end-diastolic volume from 214 ± 81 to 183 ± 79 ml (all *p* < 0.0001). Median NYHA class improved from 3 [IQR 1] (II-IV, 51/143/8) to 2 [IQR 1] (I-IV, 53/113/ 35/1) (*p* < 0.001). Reduction ≥15% of LVESV from baseline to follow-up occurred in 60% of patients, and was associated with a numerically lower hazard ratio for primary endpoint, although this reduced risk was not statistically significant (HR 0.65 [0.33–1.3], *p* = 0.21), nor for all-cause mortality; HR 0.92 [0.41–2.0], *p* = 0.83. Results were similar if LVESV indexed for body surface area was used (LVESVi, data not shown). Improvement in NYHA class occurred in 135 patients (67%), and was associated with a reduction in the hazard for the composite endpoint (HR 0.40 [0.23–0.70], *p* = 0.001), and for all-cause mortality (HR 0.26 [0.12–0.60], *p* = 0.001).

**Fig. 1:**
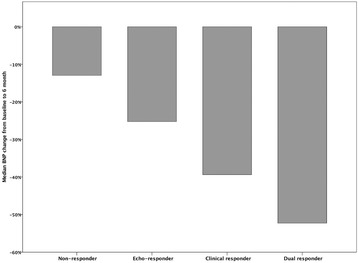
BNP reduction from baseline to 6 months, stratified for echocardiographic or clinical response. *P* values for comparisons; Clinical- vs non-responders: 0.029, Echo- vs non-responders:0.016, Double- vs non-responders: 0.011

**Fig. 2:**
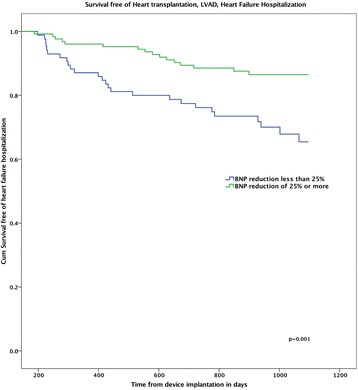
Kaplan Meier showing survival free of heart transplant and left ventricular assist device, stratified for BNP reduction ≥25%

In all there were 171 positive responders (51 clinical responders, 36 echocardiographic responders and 84 dual responders), and 31 non-responders who had neither clinical nor echocardiographic improvement. Baseline NT-proBNP levels were similar between all groups (Table 1, *p* = 0.31), and at 6 months there was an overall reduction from median 1554 [IQR 3393] to median 973 [IQR 2072], *p* < 0.001. The positive responders (regardless of subgroup) had larger reduction in NT-proBNP levels than non-responders; dual responders Δ -52%, clinical responders Δ -39%, echo-responders Δ -27% and non-responders Δ -13% (*p* = 0.01 for trend, see Fig. 1). By ROC analysis (Fig. 2), a 25% reduction of NT-proBNP was shown to have 64% sensitivity and 56% specificity for prediction of freedom of the 3-year composite endpoint in the entire cohort. In Cox regression and Kaplan Meier analysis, NT-proBNP increase, or less than 25% reduction, at 6 months was associated with higher risk of death or heart failure hospitalization (HR 0.43 [0.22–0.85], *p* = 0.02, see Fig. 2). Further ROC analyses (see Additional file 1: Figure S1) showed that including the variable for “≥25% NT-ProBNP reduction” consistently increased the area under the curve (AUC) for prediction of hard endpoints (mortality or a combination of heart failure hospitalization and mortality).

### “CRT response score”

**Fig. 3:**
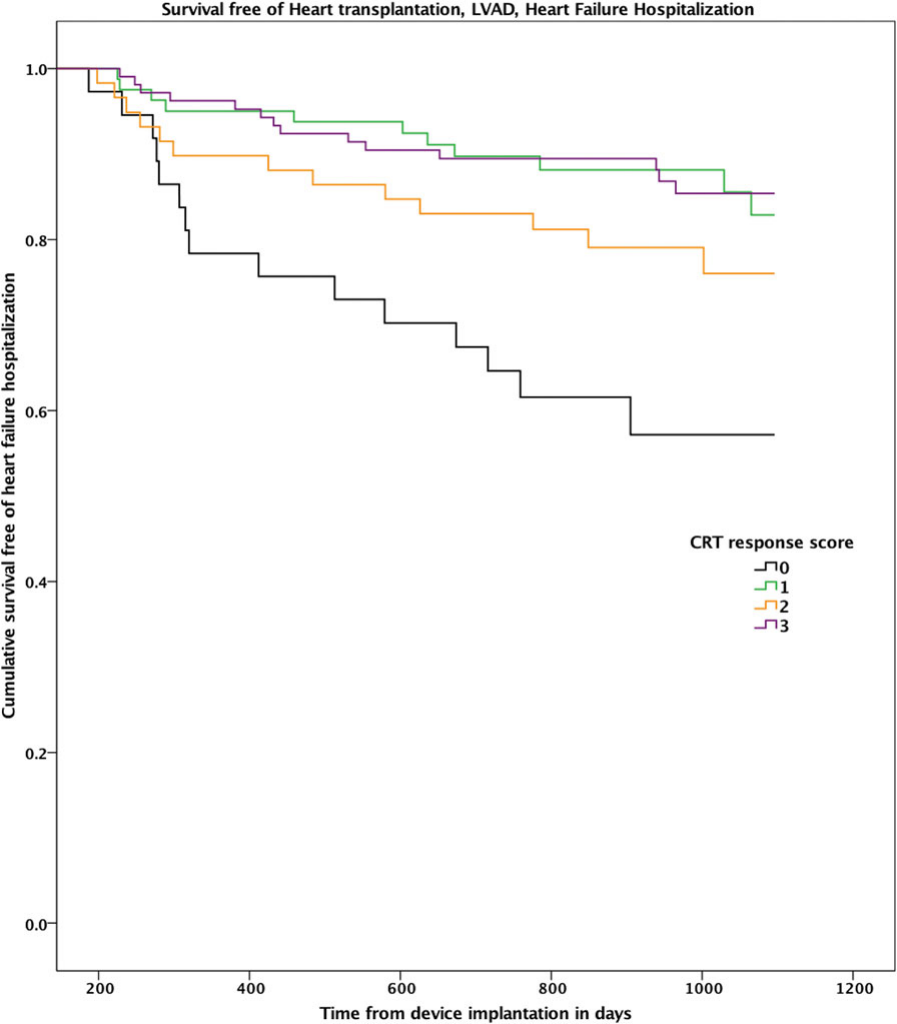
Kaplan Meier curve for survival free of heart failure hospitalization, stratified for the “CRT response score”

**Fig. 4:**
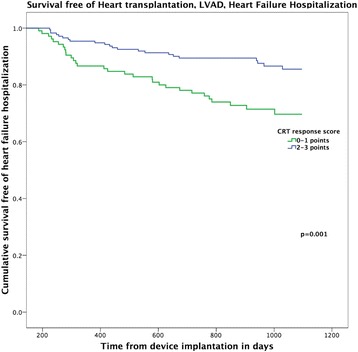
Kaplan Meier plot for survival, stratified for the “CRT response score” (0–1 points vs. 2–3 points)

A composite “CRT response score” was constructed, incorporating one point each for positive clinical (≥ 1 NYHA class improvement), echocardiographic (≥15% LVESV reduction) and biomarker (≥25% reduction in NT-proBNP) response. This score had the highest AUC value in ROC analysis for both the combined endpoint (0.67) and for the all-cause mortality endpoint (0.74). Eighteen patients (9%) had 0 points, 49 (24%) had 1 point, 75 (37%) had 2 points and 60 (30%) had 3 points on the score. In both univariate and multivariate analysis, the total CRT response score was a significant predictor the primary composite endpoint, with an independent 39% reduction of the risk of death or heart failure hospitalization for each additional point (HR 0.69 [0.50–0.96], *p* = 0.03, see Table 2). For the secondary endpoint of all-cause mortality, in multivariate analysis each additional point of the score was associated with a 52% reduction of the risk of death within 3 years (HR 0.48 [0.26–0.89], *p* = 0.02). In Kaplan Meier analysis the score was able to discriminate in particular between those patients with the lowest event-free survival (0 points), compared to patients with one or more points on the composite score who had a significantly better survival (see Fig. 3). For the all-cause mortality endpoint, those with 0–1 points had significantly higher mortality than those with 2–3 points (log rank *p* = 0.001, see Fig. 4).

**Table 2:** Cox regression analysis with three-year survival free of heart failure hospitalization as endpoint

	Univariate analysis			Multivariate analysis^a^		
	*P*-value	HR	95% C.I.	*P*-value	HR	95% C.I.
Female gender	0.12	0.44	0.15–1.2	0.24	0.59	0.24–1.4
Age, years (median IQR)	0.03	1.04	1.0–1.08	0.4	1.01	0.98–1.0
Ischemic cardiomyopathy	0.5	1.3	0.64–2.5	0.33	1.4	0.71–2.8
LV ejection fraction, (%)	0.67	0.99	0.94–1.04	0.03	0.69	0.50–0.96
Hypertension	0.79	1.1	0.55–2.2			
Diabetes	0.34	1.4	0.69–3.0			
History of atrial Fibrillation	0.22	1.5	0.78–3.0			
QRS duration	0.32	0.99	0.98–1.0			
Left bundle branch block	0.1	0.57	0.29–1.1	0.03	0.51	0.28–0.93
ACEi or ARB use	0.5	0.72	0.28–1.9			
Loop diuretic use	0.34	1.5	0.66–3.5			
Beta-blocker use	0.4	1.7	0.51–5.4			
Creatinine pre implant	0.49	1.1	0.83–1.5			
CRT-P (compared to CRT-D)	0.02	2.4	1.2–5.1	0.21	1.6	0.78–3.2
NT-proBNP baseline (per 100 ng/L)	0.02	1.01	1.001–1.009			
NYHA class baseline	0.52	0.85	0.52–1.4			
Composite score (per point)	< 0.0001	0.52	0.36–0.74	0.03	0.69	0.50–0.96

*CRT* cardiac resynchronization therapy, *ACEi* ACE-inhibitor, *ARB* angiotensin receptor blocker, *CRT-D* cardiac resynchronization therapy with defibrillator function, *CRT-P* cardiac resynchronization therapy without defibrillator function

^*^Multivariate model corrected for gender, age, LBBB, type of cardiomyopathy and type of CRT-device

## Discussion

In this prospective two-center cohort of consecutive CRT treated patients, we show that by combining echocardiographic, clinical and biomarker information into a simple 0–3 point score, we were able to identify the patients with the highest risk of adverse clinical outcome. This may have implications for how to set up a structured follow-up of CRT implanted patients; by using the proposed simple scoring system, the clinician will be able to identify most patients with poor prognosis at the 6-months follow-up visit, thus enabling a more focused intervention to try to potentially convert these patients to responders and/or improve their clinical outcome [24, 25].

### Echocardiography based parameters

Several measures of “response” have been used in the randomized trials. The MADIT-CRT trial was the first to report more extensively on the effect of echocardiographic remodeling measures on subsequent clinical outcome [26, 27]. The authors showed that end-systolic volume, end-diastolic volume and LVEF were all correlated to decreased hazard of the long-term composite clinical endpoint (with the same magnitude of hazard ratio from 0.60–0.72 for a 10% relative decrease in volume or a 5% absolute increase in LVEF). The REVERSE study showed similar results with a 14% hazard reduction of 5-year mortality for every 10% decrease in LVESV-index, and a 68% risk reduction for a dichotomized endpoint of LVESVi decrease≥15% [28]. On the contrary, our results from the present non-randomized observational cohort do not show a strong correlation between echocardiographic remodeling and clinical outcome. This may be due to reduced statistical power because of relatively few patients in the study, since the non-significant hazard ratio of 0.65 was of similar magnitude as in the randomized trials, and indicated a trend in the same direction. Since the established correlation between reverse remodeling and later reduction in clinical endpoints is strong, this prompted us to keep the echocardiographic evaluation in the final composite score. As was seen in the REVERSE trial, it may take longer for the CRT induced remodeling evident at 6 months post-implant to have an effect on actual clinical outcome [29, 30].

### Clinical improvement

The “Packer clinical score” has been used in several CRT studies and other studies on heart failure patients, and has been shown to correlate with clinical outcome [31]. The most widely used definition in the early CRT studies was improvement in NYHA class ≥1 from baseline to follow-up [32, 33], but other subjective patient-based evaluations have also been shown to correlate to clinical outcome [34]. The evaluation of clinical status is prone to placebo-effect on the part of the patient, and has its limitations with regards to inter-individual reproducibility on the part of the physician. However, it is an important factor in the follow-up for CRT treated patients, since many patients (and physicians) would be unwilling to make major changes to an ongoing treatment when the patient experiences a definite improvement compared to the pre-operative status. In line with many previous studies, we show that improvement in NYHA class is strongly associated (HR 0.21 for all-cause mortality and HR 0.53 for the composite endpoint) to better clinical outcome, and an important parameter in the comprehensive clinical evaluation of CRT response.

### Role of NT-ProBNP

BNP and NT-proBNP levels are increased in HF, and correlate well with ventricular wall stress and severity of HF. Elevation of these peptides has a well-established role for prediction of mortality for both acute and chronic heart failure situations [35, 36]. In the CRT setting, the evidence for baseline BNP levels to predict clinical outcome is ambiguous; higher levels of BNP pre-implant have been correlated to higher risk of 1 year adverse events post-implant by some investigators [37, 38], but other studies have not shown any correlation of baseline BNP to CRT-response [39]. Most likely this is due to the fact that successful CRT treatment can occur regardless of baseline BNP levels, and depends on a number of factors unassociated with baseline BNP levels, hence the resynchronization effect (and clinical outcome) is hard to predict by this metric. However, a *decrease* in BNP or NT-proBNP has consistently been showed to correlate with successful resynchronization and improvement of heart failure symptoms in CRT trials [40–42]. In those with longer follow-up times, this BNP reduction also transformed into reduced risk of adverse events and mortality [43, 44]. In the MADIT-CRT trial, patients who received CRT-T and at 1 year post-implant had reduced BNP-values (or low values at baseline that remained unchanged at follow-up), had significantly lower risk of subsequent heart failure hospitalization or death [15]. In the present study we found that BNP levels were significantly reduced in responders (echo- or clinical) compared to non-responders, and that the greatest reduction was seen in those patients who exhibited both a clinical and echocardiographic positive response. In contrast, patients who did not respond on any of these three parameters, had a poor prognosis with significantly higher mortality and heart failure hospitalizations. A cutoff of 25% reduction in BNP was associated with better clinical outcome, and proved to be useful in combination with the other parameters in the CRT response score. However, the sensitivity and specificity were not good enough for a specific cut-off value for NT-ProBNP reduction to be used as a stand-alone criterion for accurate prediction of clinical outcome.

### Clinical utility of the CRT response score

The combined score had an independent predictive value for clinical outcome; each additional point of the score was associated with a 52% reduction of the risk of death within 3 years (HR 0.48 [0.26–0.89], *p* = 0.02), but for practical purposes the major prognostic difference was for those patients who did not fulfill any of the three response criteria. This was visible in Kaplan Meier analysis where there was a clear separation of the patients with zero points compared to patients with one or more points regarding mortality and hear failure hosopitalization outcome. Indeed, the minority group of patients with zero CRT response score points had an event-free 3-year survival of less than 60%, and therefore could potentially benefit from intensified care or even reoperation in case of suboptimal CRT effect. Assessing clinical response to CRT can be challenging, especially when having the placebo effect in mind. Using a natriuretic peptide for a more objective evaluation of decompensated left ventricular function can therefore be helpful. Thus, adding an NT-proBNP test to the preoperative and post-implant evaluation is an inexpensive tool that has the potential to aid the clinician in making an informed decision on which patients have a poor prognosis and need to be more thoroughly evaluated. Our findings add to the present evidence for the important role of dynamic changes in BNP /NT-proBNP in relation to clinical outcome in CRT.

Other factors, including diabetes and gender, have by some been suggested as prognostic factors in CRT treated patients, but analyses in our cohort showed no significant difference in prognosis for these parameters, and our results are in line with previous data suggesting that diabetes and gender do not limit CRT response [45].

### Limitations

This study included a limited number of patients, from two tertiary care institutions. Even though the baseline demographics indicate that the patient cohort is similar to most of the previously published studies, there may be a selection bias compared to the general “real life” CRT patients seen at other non-tertiary referral implanting centers. The echocardiography data were analyzed at two different locations, and there may be differences between the echocardiography readings that are not accounted for. Data on LV electrode position, and % of biventricular pacing post-implant were not included in the analysis, but every effort was made to ensure ≥95% biventricular pacing, and the implanting physicians uniformly targeted the LV lead placement in a lateral or posterolateral position with long electrical delay (measured as interlead RV-LV delay). Furthermore, the implants took place prior to the widespread use of quadripolar leads, and only one patient in the cohort had a quadripolar lead implanted. This may have influenced the proportion of CRT responders negatively. Echocardiographic optimization was performed at the patients in one of the two institutions, whereas the other institution used an EGM-based optimization (Quickopt®) built into the device as standard, a method which has been validated and found similar to echocardiography-based optimization [46]. This may have introduced a bias, even though there was no significant difference in clinical outcome between the two centers. The NT-ProBNP blood samples were drawn in an ambulatory setting with the patient in stable cardiac condition, but the patient’s clinical status was not evaluated at the same day, and therefore a potential confounding of results due to intermittent fluid overload or concurrent infection etc. cannot be ruled out. The percentage of women in this study was relatively low, which affects the generalizability of the results to women with HF. The cluster of risk factors called metabolic syndrome has also been suggested as a prognostic factor for CRT patients, but unfortunately we were not able to investigate this since our data did not include incidence of the metabolic syndrome. No continuous remote monitoring of the patients was performed during the follow up period.

## Conclusion

Post-implant changes in NT-proBNP correlate with echocardiographic and clinical response to CRT. By using a combined “CRT response score”, consisting of echo-, clinical-, and biochemical response criteria, 3-year clinical outcome can be predicted with higher accuracy. This information may be particularly helpful for a better identification of non-responders with poor clinical outcome, and could direct attention to the patients in need of a re-intervention or modification of relevant CRT-related parameters.

## Additional file

Additional file 1: **Figure S1.** ROC analysis for change in NT- proBNP vs. freedom from composite endpoint at 3 years. (JPEG 1805 kb)

## Abbreviations

ACEi: Angiotensin converting enzyme inhibitor
AF: Atrial fibrillation
ARB: Angiotensin receptor blocker
BNP: Brain natriuretic peptide
CABG: Coronary artery bypass surgery
CRT: Cardiac Resynchronization therapy
CRT-D: Cardiac Resynchronization therapy with defibrillator function
CRT-P: Cardiac Resynchronization therapy without defibrillator function
CS: coronary sinus
EF: Ejection fraction
HR: Hazard ratio
LBBB: Left bundle branch block
LV: Left ventricle
LVAD: Left ventricular assist device
LVEDD: Left Ventricular end diastolic diameter
LVEDV: Left Ventricular end diastolic volume
LVESV: Left Ventricular end systolic volume
LVESVi: Left Ventricular end systolic volume index
NT-proBNP: N terminal pro Brain Natriuretic Peptide
NYHA: New York Heart Association
RV: Right ventricle
