# Supplementary figures and images for: Prediction of clinical outcome in patients treated with cardiac resynchronization therapy - the role of NT-ProBNP and a combined response score
Source: BMC Cardiovasc Disord. 2018 Apr 24;18:70. doi: 10.1186/s12872-018-0802-8 (PMC5921413; doi:10.1186/s12872-018-0802-8)

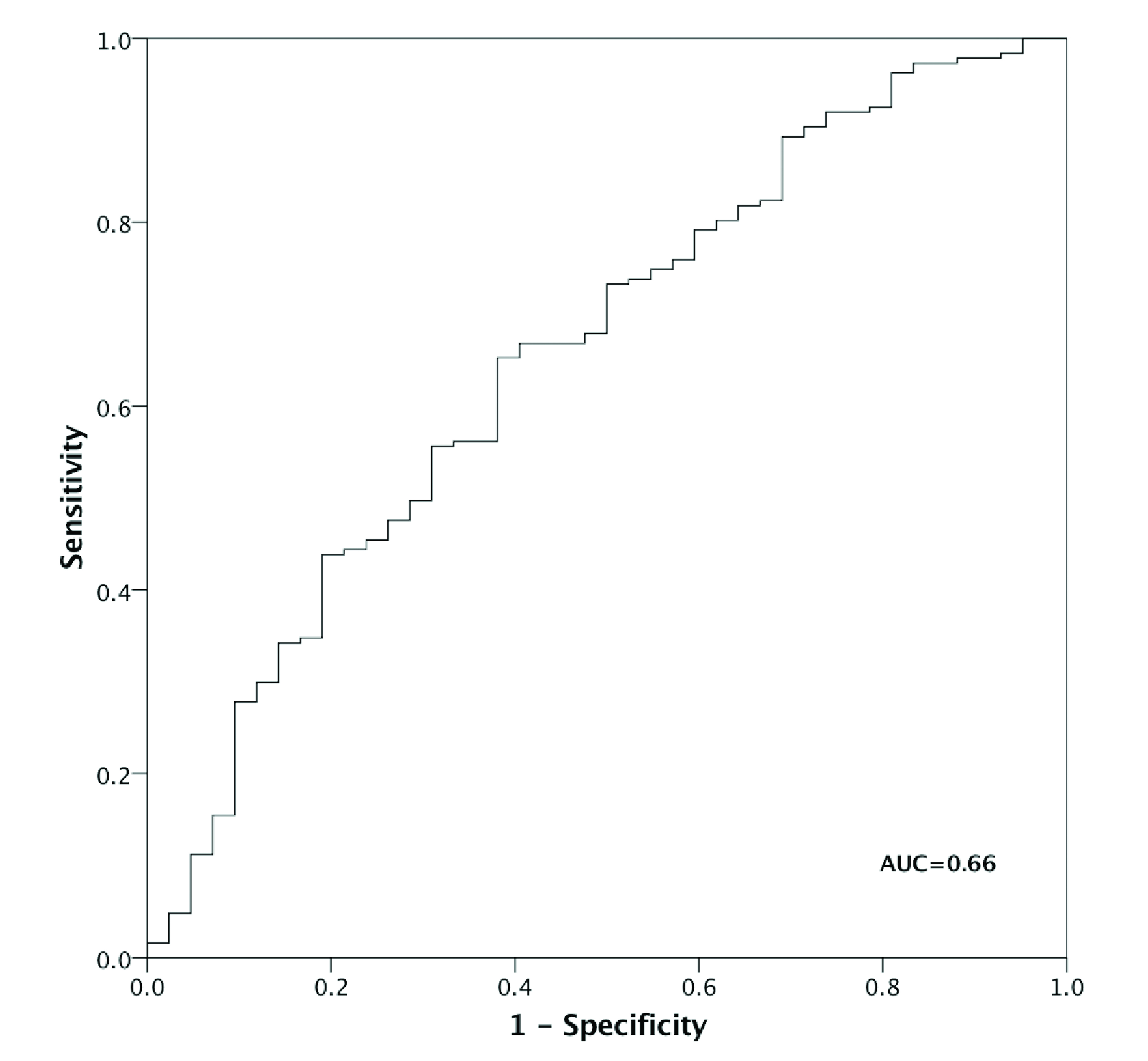

Supplement: Supplementary file 1 — Figure S1. ROC analysis for change in NT- proBNP vs. freedom from composite endpoint at 3 years. (JPEG 1805 kb) [file 12872_2018_802_MOESM1_ESM.jpg]
